# Geniposide Improves Diabetic Nephropathy by Enhancing ULK1-Mediated Autophagy and Reducing Oxidative Stress through AMPK Activation

**DOI:** 10.3390/ijms22041651

**Published:** 2021-02-06

**Authors:** Theodomir Dusabimana, Eun Jung Park, Jihyun Je, Kyuho Jeong, Seung Pil Yun, Hye Jung Kim, Hwajin Kim, Sang Won Park

**Affiliations:** 1 Department of Pharmacology, Institute of Health Sciences, Gyeongsang National University College of Medicine, Jinju 52727, Korea; odomy2020@gmail.com (T.D.); foreverpak1@nate.com (E.J.P.); jeri1984@naver.com (J.J.); khjeong@gnu.ac.kr (K.J.); spyun@gnu.ac.kr (S.P.Y.); hyejungkim@gnu.ac.kr (H.J.K.); 2 Department of Convergence Medical Sciences, Institute of Health Sciences, Gyeongsang National University Graduate School, Jinju 52727, Korea

**Keywords:** diabetic nephropathy, geniposide, autophagy, inflammation, oxidative stress, AMPK

## Abstract

Diabetic nephropathy (DN) is a common pathological feature in patients with diabetes and the leading cause of end-stage renal disease. Although several pharmacological agents have been developed, the management of DN remains challenging. Geniposide, a natural compound has been reported for anti-inflammatory and anti-diabetic effects; however, its role in DN remains poorly understood. This study investigated the protective effects of geniposide on DN and its underlying mechanisms. We used a C57BL/6 mouse model of DN in combination with a high-fat diet and streptozotocin after unilateral nephrectomy and treated with geniposide by oral gavage for 5 weeks. Geniposide effectively improves DN-induced renal structural and functional abnormalities by reducing albuminuria, podocyte loss, glomerular and tubular injury, renal inflammation and interstitial fibrosis. These changes induced by geniposide were associated with an increase of AMPK activity to enhance ULK1-mediated autophagy response and a decrease of AKT activity to block oxidative stress, inflammation and fibrosis in diabetic kidney. In addition, geniposide increased the activities of PKA and GSK3β, possibly modulating AMPK and AKT pathways, efficiently improving renal dysfunction and ameliorating the progression of DN. Conclusively, geniposide enhances ULK1-mediated autophagy and reduces oxidative stress, inflammation and fibrosis, suggesting geniposide as a promising treatment for DN.

## 1. Introduction

Diabetic nephropathy (DN) is one of the most common microvascular complications of type 1 and type 2 diabetes and the leading cause of chronic kidney disease (CKD) and end-stage renal disease (ESRD) [1,2]. DN is characterized by a decreased glomerular filtration rate (GFR), albuminuria and elevated plasma creatinine. Early stages of DN exhibit podocyte loss, glomerular hypertrophy, mesangial matrix expansion and glomerular basement membrane thickening; while advanced stages of DN exhibit nodular glomerulosclerosis, mesangiolysis, enhanced inflammation and tubulo-interstitial fibrosis [3,4]. Intensive glycemic and blood pressure (BP) control or renin-angiotensin-aldosterone system (RAAS) inhibition is the currently available treatment for patients with DN; however, many patients still progress to CKD, resulting to ESRD [5]. Therefore, there is an emergent need for understanding the precise pathologic mechanisms of DN and developing novel therapeutic agents.

Autophagy is a highly conservative cellular process that degrades and recycles misfolded or dysfunctional proteins and damaged organelles to maintain cellular homeostasis via the lysosome pathway [6,7]. Autophagy is regulated through the nutrient-sensing signaling pathways that are often altered in diabetes due to metabolic stress [8]. Previously, autophagy deficiency induces glomerular and tubular cell injury in experimental animal models of DN, contributing to the pathogenesis of DN [9,10]. Therefore, restoration of autophagy activity is considered as a promising therapeutic target to prevent the progression of DN.

AMP-activated protein kinase (AMPK), a nutrient-sensing kinase, is activated by phosphorylation in the metabolic process of fatty acid oxidation, glucose uptake and glycolysis at low energy states. AMPK has shown to positively activate autophagy, and the reduced AMPK has shown to be involved in the pathogenesis of DN [9]. Pharmacological activation of AMPK by 5-Aminoimidazole-4-carboxamide ribonucleotide (AICAR), metformin or resveratrol significantly improves glomerular and tubular injury in diabetic animal models [9,11,12,13]. Importantly, AMPK promotes autophagy by directly activating Unc-51 like autophagy activating kinase 1 (ULK1) through phosphorylation of Ser317 and Ser777 under glucose starvation. In contrast, under nutrient sufficiency, high mTORC1 activity prevents ULK1 activation by phosphorylating ULK1 at Ser757 and disrupting the interaction between ULK1 and AMPK, thereby reducing AMPK-ULK1 signaling-mediated autophagy induction [14]. In addition, the activation of AMPK can phosphorylate tuberous sclerosis complex 2 (TSC2) and the activated TSC2 can suppress mTORC1 to induce autophagy [15]. A recent study has reported that AMPK also contributes to autophagosome maturation and lysosome fusion in HEK293 cells [16]. Thus, AMPK is a critical regulator of autophagy and AMPK-induced autophagy is renoprotective mechanism of DN. 

Geniposide is an iridoid glycoside compound, one of the active ingredients extracted from the fruits of Gardenia jasminoides Ellis, which have long been used in traditional Chinese medicine [17]. This compound has primarily shown pharmacological effects of anti-inflammatory [18], anti-oxidative [19], neuroprotective [20], anti-angiogenic [21] and anti-diabetic properties [22,23]. Particularly, geniposide reduces renal inflammation via regulating nuclear factor kappa B (NFkB) signaling in streptozotocin (STZ)-induced diabetic rats [22]. Geniposide also shows a hypoglycemic effect by inhibiting glycogen phosphorylase and glucose-6-phosphatase in type 2 diabetic mice, induced by a high-fat diet (HFD) and STZ injection [23]. Geniposide protects β-cell survival in cultured mouse islets and promotes β-cell regeneration to normalize blood glucose in HFD and db/db mice [24]. A recent study reported that geniposide blocks oxidative stress and inflammatory response, inhibiting podocyte pyroptosis, thereby alleviating renal pathology in HFD/STZ-induced DN mice [25]. 

Considering that autophagy is an important therapeutic target for DN, we investigated whether geniposide modulates autophagy activity in DN and explored the underlying molecular mechanisms. In this study, we demonstrated that geniposide enhanced ULK1-mediated autophagy response and reduced oxidative stress through AMPK activation, thus ameliorating glomerular and tubular injury, inflammation and interstitial fibrosis induced by DN.

## 2. Results

### 2.1. Geniposide Improved Glomerular Filtration Function and Structural Lesions in DN

**Figure 1:**
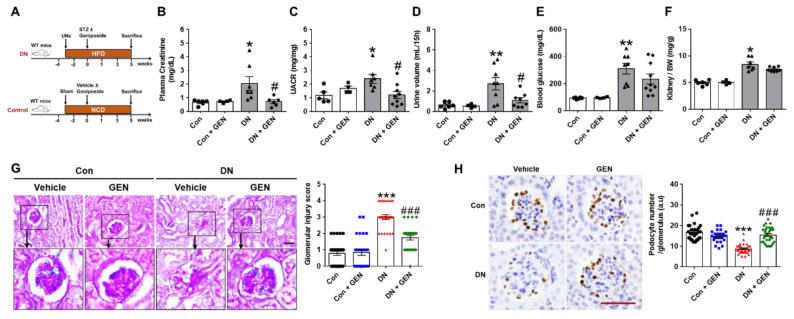
Geniposide decreases albuminuria and renal pathology in diabetic nephropathy (DN). (**A**) DN mouse model was generated by streptozotocin (STZ) injection (100 mg/kg) to the mice after 3 weeks of unilateral nephrectomy (UNx) and high-fat diet (HFD) feeding. After STZ, mice were administered with geniposide (50 mg/kg) or vehicle by oral gavage for 5 weeks. The control mice were fed with a normal chow diet (NCD) and received vehicle or geniposide. At sacrifice, the blood and urine samples were collected from each group. (**B**) Plasma creatinine, (**C**) urine albumin to creatinine ratio (UACR), (**D**) urine volume (mL/15 h), (**E**) blood glucose and (**F**) kidney/body weight (BW) ratio were analyzed, respectively (Control (Con) or Control + geniposide (Con + GEN), *n* = 4–6; DN or DN + GEN, *n* = 7–10). (**G**) Kidney sections were processed for PAS staining, representative images were shown, and glomerular morphological changes were scored, as described in the method (*n* = 3–5). (**H**) Kidney sections were processed for WT1 immunostaining; representative images are shown and the number of stained podocytes per glomerulus was counted using ImageJ software (*n* = 3–5). Data are presented as the mean ± SEM. One-way ANOVA was used for statistical analysis followed by Bonferroni’s multiple comparisons. * *p* < 0.05, ** *p* < 0.01, *** *p* < 0.001 vs. control mice; and ^#^ *p* < 0.05, ^###^ *p* < 0.001 vs. DN mice. Scale bar, 50 µm.

To determine the effect of geniposide on the renal pathology of DN, we evaluated the parameters of renal injury in control and DN mice administered with vehicle or geniposide (50 mg/kg) for 5 weeks (Figure 1A). DN mice treated with geniposide markedly reduced plasma creatinine, urine albumin to creatinine ratio (UACR) and the excreted urine volume (Figure 1B–D) compared to DN mice. However, plasma blood glucose and relative kidney weight had no statistical differences in DN mice treated with geniposide compared to DN mice (Figure 1E,F). There was also no difference in body weight between DN and DN mice treated with geniposide (data not shown). Next, we determined the extent of histopathological changes by PAS staining and found that DN mice showed different degrees of hyalinization, glomerular atrophy, reduced Bowman’s capsule space and chronic glomerulonephritis; however, these features of glomerular injury were significantly reduced by geniposide treatment (Figure 1G). Consistently, the podocyte number assessed by WT1 immunostaining was significantly reversed by geniposide treatment in DN mice, clearly indicating the protective effect of geniposide on podocyte loss (Figure 1H). These results suggest a potential renoprotective effect of geniposide that improves glomerular filtration function and structural lesions in DN. 

### 2.2. Geniposide Reduced Renal Tubular Injury and Interstitial Fibrosis in DN

**Figure 2:**
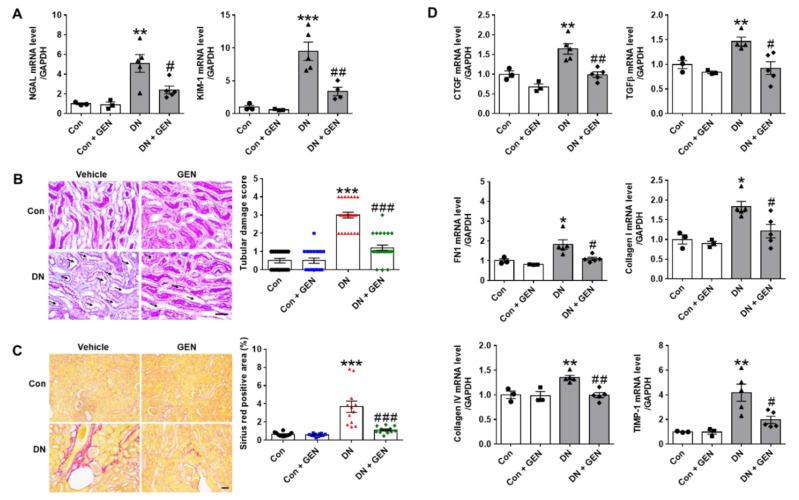
Geniposide reduces renal tubular injury and interstitial fibrosis in DN. (**A**) Relative mRNA levels of kidney injury molecule-1 (KIM-1) and neutrophil gelatinase-associated lipocalin (NGAL), specific biomarkers for renal tubular injury, were assessed by real-time PCR analysis (*n* = 3–5). (**B**) Renal tubular injury was also analyzed by PAS staining, the representative images were shown, and the tubular damage scores were evaluated (black arrows indicate damaged tubules), (*n* = 3–5). (**C**) Kidney sections were processed for Picro-Sirius Red staining and fibrotic area was presented as stained areas in percentages (%) using ImageJ software (*n* = 3–5). (**D**) Relative mRNA levels of fibrogenic genes were determined by real-time PCR analysis. Relative mRNA expression was normalized to that of GAPDH (*n* = 3–5). Data are presented as the mean ± SEM. One-way ANOVA was used for statistical analysis followed by Bonferroni’s multiple comparisons. * *p* < 0.05, ** *p* < 0.01, *** *p* < 0.001 vs. control mice; and ^#^ *p* < 0.05, ^##^ *p* < 0.01, ^###^ *p* < 0.001 vs. DN mice. Scale bar, 50 µm.

KIM-1 (kidney injury molecule-1) and NGAL (neutrophil gelatinase-associated lipocalin) are recently considered as sensitive biomarkers of tubular injury in acute kidney injury (AKI) as well as CKD [26,27]. To assess the effect of geniposide on tubular damage, the expression of these biomarkers was evaluated by real-time PCR analysis. Consistent with our previous findings [28], the mRNA levels of KIM-1 and NGAL were significantly upregulated in DN mice compared to control, but reduced by geniposide treatment (Figure 2A). The protective effect of geniposide was further confirmed by PAS staining; DN mice showed a significant increase of tubulo-interstitial damage including tubular dilatation, loss of brush borders, necrosis and extracellular accumulation, which was reduced by geniposide treatment (Figure 2B, damaged tubules are indicated by black arrows). These features of tubular injury are mutually induced with interstitial fibrosis and exacerbates the pathology of DN [29]. Therefore, we examined the effect of geniposide on interstitial fibrosis by Picro-Sirius Red staining and real-time PCR analysis. Geniposide treatment significantly reduced collagen deposition in the kidney of DN mice (Figure 2C). Consistently, the mRNA levels of fibrogenic mediators including connective tissue growth factor (CTGF), transforming growth factor β (TGFβ), fibronectin 1 (FN1), collagen I and IV and tissue inhibitor of metalloproteinase-1 (TIMP-1) were significantly upregulated in DN mice compared to control; but geniposide treatment significantly reduced their expression. (Figure 2D). Collectively, the results suggest that geniposide treatment has a renoprotective effect by reducing renal tubular injury and interstitial fibrosis in DN. 

### 2.3. Geniposide Decreased Renal Apoptosis and Oxidative Stress in DN

**Figure 3:**
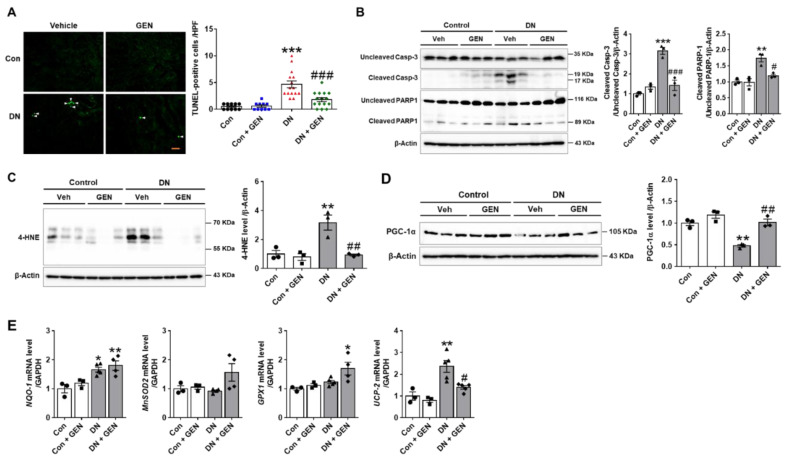
Geniposide decreases renal apoptosis and oxidative stress in DN. (**A**) Representative images of TUNEL staining processed in the kidney sections to determine DN-induced renal apoptosis. The number of TUNEL-positive cells/high power field (HPF) was counted to present the severity of apoptosis (*n* = 3). (**B**) The extent of caspase-3 and PARP-1 cleavage was determined in the kidney tissue lysates by western blot analysis and the relative quantification was shown (*n* = 3). (**C**) Oxidative stress was assessed by measuring 4-HNE, a marker of lipid peroxidation by western blot analysis and the relative quantification was shown (*n* = 3). (**D**) The protein expression levels of PGC-1α and β-actin, as a loading control, were determined by western blot analysis in kidney tissue lysates. Quantitative analysis was shown (*n* = 3). (**E**) The Relative mRNA levels of NQO-1, MnSOD2, GPX1 and UCP2 were determined by real-time PCR analysis. Relative mRNA expression was normalized to that of GAPDH (*n* = 3–5). Data are presented as the mean ± SEM. One-way ANOVA was used for statistical analysis followed by Bonferroni’s multiple comparisons. * *p* < 0.05, ** *p* < 0.01, *** *p* < 0.001 vs. control mice; and ^#^ *p* < 0.05, ^##^ *p* < 0.01, ^###^ *p* < 0.001 vs. DN mice. Scale bar, 50 µm.

Renal apoptosis and oxidative stress are the common pathological features involved in the progression of DN in experimental mouse models and human patients [30,31,32]. To investigate the effect of geniposide on renal apoptosis induced by DN, we performed TUNEL staining to determine the extent of apoptosis. The number of TUNEL-positive cells was significantly increased in DN mice compared to control, but reduced by geniposide treatment (Figure 3A). We also conducted the western blot analysis to assess cleavage of caspase-3 and PARP-1. The cleaved caspase-3 and PARP-1 were markedly increased in DN mice but were reversed by geniposide treatment (Figure 3B). Furthermore, oxidative stress assessed by 4-HNE, a marker of lipid peroxidation, was significantly upregulated in DN mice and was inhibited upon geniposide treatment (Figure 3C). We also determined the expression of peroxisome proliferator activated receptor γ (PPARγ) coactivator-1α (PGC-1α), a master regulator of mitochondria biogenesis. PGC-1α is a transcription factor associated with high metabolic activity in many tissues, but also involved in the induction of antioxidant genes upon stress [33,34]. Geniposide treatment normalized the reduced expression of PGC-1α in DN to control levels, consistently increased the mRNA levels of antioxidant gene including NAD(P)H quinone oxidoreductase-1 (NQO-1), manganese superoxide dismutase (MnSOD2) and glutathione peroxidase-1 (GPX-1) (Figure 3D,E). In addition, the mRNA levels of uncoupling protein-2 (UCP-2), localized in the inner mitochondrial membrane acting as a protonophore, were downregulated by geniposide treatment (Figure 3E), blocking deleterious effects of UCP2 in DN [35]. The results indicate that geniposide effectively suppresses renal apoptosis and oxidative stress induced by DN. 

### 2.4. Geniposide Suppressed Macrophage Infiltration and Renal Inflammation in DN 

**Figure 4:**
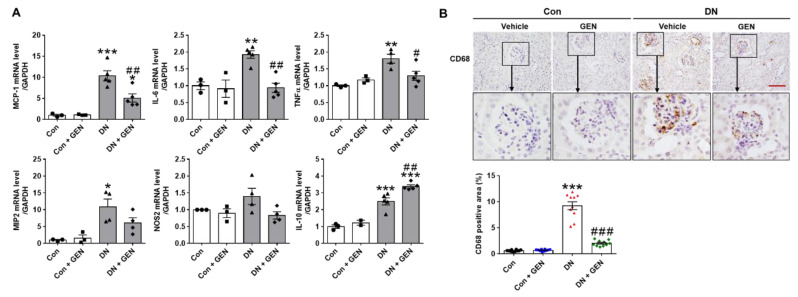
Geniposide decreases renal inflammation and macrophage infiltration in DN. (**A**) Relative mRNA levels of pro-inflammatory cytokines (TNFα, IL-6, NOS2), chemokines (MCP-1, MIP2) and anti-inflammatory cytokine (IL-10) were determined by real-time PCR analysis. Relative mRNA expression was normalized to that of GAPDH (*n* = 3–5). (**B**) Kidney sections were processed for immunostaining of CD68, a macrophage marker, and the representative images were shown. The CD68-positive area (%) was calculated using ImageJ software (*n* = 3). Data are presented as the mean ± SEM. One-way ANOVA was used for statistical analysis followed by Bonferroni’s multiple comparisons. * *p* < 0.05, ** *p* < 0.01, *** *p* < 0.001 vs. control mice; and ^#^ *p* < 0.05, ^##^ *p* < 0.01, ^###^ *p* < 0.001 vs. DN mice. Scale bar, 50 µm.

Macrophage infiltration into glomeruli and the increased pro-inflammation cytokines during early stages of DN aggravate pathological features of DN [36,37]. We first, investigated whether geniposide treatment affects pro-inflammatory cytokine and chemokine expression using real-time PCR analysis. Notably, DN increased the mRNA levels of pro-inflammatory cytokines (tumor necrosis factor α (TNFα), interleukin 6 (IL-6) and inducible nitric oxide synthase 2 (NOS2)) and chemokines (monocyte chemoattractant protein-1 (MCP-1) and macrophage inflammatory protein 2 (MIP2)); while geniposide treatment downregulated these changes. Geniposide also enhanced anti-inflammatory cytokine expression of IL-10 (Figure 4A). The renal induction of MCP-1 involved in the recruitment of macrophage and other inflammatory cells contributes to macrophage infiltration in diabetic kidneys [38]. Thus, we examined CD68, a marker of macrophage, by IHC staining. The results showed that CD68-positive cells were significantly infiltrated into glomeruli in DN mice compared to control; however, geniposide treatment markedly inhibited macrophage infiltration in DN mice (Figure 4B). The results suggest that geniposide treatment attenuates macrophage infiltration and renal inflammation in DN. 

### 2.5. Geniposide Increased Autophagy Response in DN 

**Figure 5:**
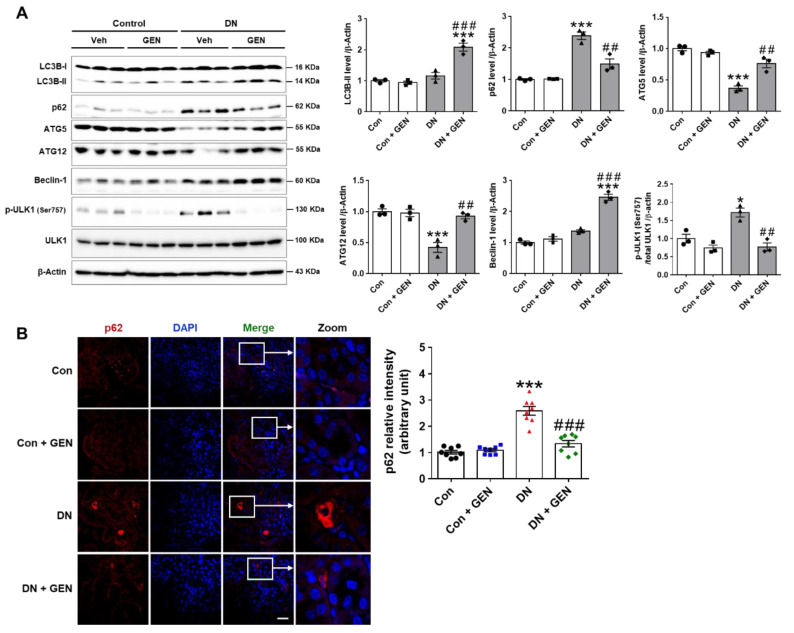
Geniposide enhances autophagy response in DN. (**A**) Kidney tissues were lysed for western blot analysis and the levels of autophagy-related proteins (LC3B, p62, ATG5, ATG12, Beclin-1 and ULK1) and β-actin, as a loading control, were determined. Quantitative analysis of each protein was shown (*n* = 3). (**B**) Kidney sections were processed for p62 immunofluorescence staining and the representative images were shown. The relative intensity of p62 was analyzed using Olympus Fluoview FV1000 software (Olympus), (*n* = 3). Data are presented as the mean ± SEM. One-way ANOVA was used for statistical analysis followed by Bonferroni’s multiple comparisons. * *p* < 0.05, *** *p* < 0.001 vs. control mice; and ^##^ *p* < 0.01, ^###^ *p* < 0.001 vs. DN mice. Scale bar, 50 µm.

Autophagy deficiency in diabetic kidneys contributes to the development and progression of DN [10]. Pharmacological activation of autophagy is a promising therapeutic option that improves the pathogenesis of DN, as shown in our previous work and other studies [13,32,39]. To determine the effect of geniposide on autophagy in DN, we examined the renal expression of autophagy-related proteins (ATGs), Beclin-1, autophagy flux markers (LC3B and p62), and ULK1 phosphorylation. Geniposide treatment significantly enhanced the expression of ATG5, ATG12 and Beclin-1, and improved autophagy flux as determined by the increased LC3B-II and decreased p62 levels compared to DN mice. The inhibitory phosphorylation of ULK1 at Ser757 was decreased by geniposide treatment, indicating that ULK1-mediated autophagosome formation was restored by geniposide (Figure 5A). We also performed the immunofluorescence staining of p62; impaired autophagy is evidenced by cytoplasmic accumulation of p62, a substrate degraded through the autophagy-lysosomal pathway. A significant cytoplasmic accumulation of p62 was found in DN mice compared to control, which was reduced upon geniposide treatment (Figure 5B). These results suggest that geniposide treatment enhances autophagy activity and possibly ameliorates the progression of DN.

### 2.6. Geniposide Protected the Kidney through AMPK Activation and AKT Inhibition in DN

**Figure 6:**
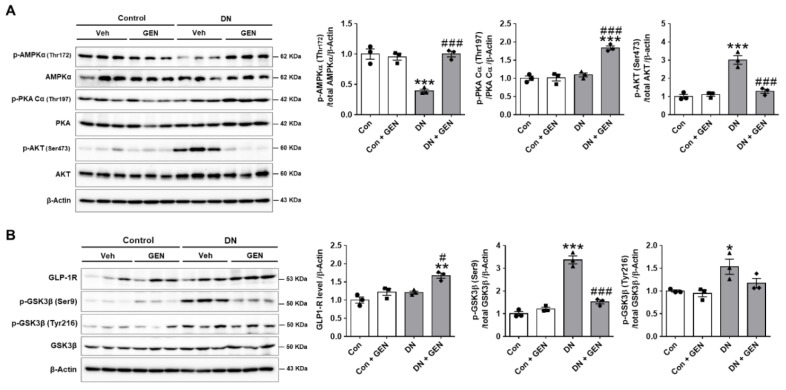
Geniposide protects the kidney through AMPK activation and AKT inhibition in DN. (**A**) The protein expression levels of p-AMPKα (Thr172), p-PKA Cα (Thr197), p-AKT (Ser473), and β-actin, as a loading control, were determined by western blot analysis in kidney tissue lysates. Quantitative analysis of each protein level was shown (*n* = 3). (**B**) The protein expression levels of GLP-1R, p-GSK3β (Ser9), p-GSK3β (Tyr216), GSK3β and β-actin, as a loading control, were determined by western blot analysis in kidney tissue lysates. Quantitative analysis was shown (*n* = 3). Data are presented as the mean ± SEM. One-way ANOVA was used for statistical analysis followed by Bonferroni’s multiple comparisons. * *p* < 0.05, ** *p* < 0.01, *** *p* < 0.001 vs. control mice; and ^#^ *p* < 0.05, ^###^ *p* < 0.001 vs. DN mice.

To investigate the potential protective mechanism of geniposide in DN, the expression of p-AMPK, p-PKA and p-AKT, known to regulate autophagy response, was examined by western blot analysis. Given that autophagy plays a crucial role in maintaining the homeostasis of podocytes and proximal tubular cells, an increased activity of AMPK is reported to activate autophagy and attenuates the pathogenesis of DN [40]. Moreover, PKA, an upstream kinase, modulates the AMPK activity to attenuate renal dysfunction in CKD [41]. Consistently, geniposide treatment significantly increased p-AMPK and p-PKA in DN (Figure 6A), suggesting that these kinase activities play a protective role against DN. In response to stress or pathological conditions, AKT phosphorylates mTORC1, which in turn represses autophagy by phosphorylating ULK1 at Ser757, inhibiting the ULK1-mediated initiation of autophagy; conversely, AMPK activates autophagy through phosphorylating ULK1 at Ser777, which suggests that AMPK may affect AKT-ULK1 interaction [40,42,43]. Thus, we examined whether geniposide treatment affects p-AKT expression in DN. The results showed that p-AKT levels upregulated in DN were significantly decreased by geniposide (Figure 6A), which correlates with activation of ULK1 to restore autophagy function. In addition, we investigated whether geniposide affects the GSK3β activity in DN, as previous studies report differential effects in the regulation of autophagy by GSK3β, and associated pathology in diabetic kidneys [44,45,46]. Our results showed that GSK3β was significantly activated by geniposide treatment in DN mice, as indicated by reduced levels of inactive p-GSK3β (Ser9); but there was no significant change in the levels of active p-GSKβ (Tyr216) (Figure 6B). Taken together, geniposide treatment enhances autophagy activity through ULK1-mediated pathway by AMPK activation and AKT inhibition, thereby ameliorating the development and progression of DN. 

### 2.7. Protective Mechanism of Geniposide against DN by Improving Autophagy and Inhibiting Oxidative Stress through AMPK Activation and AKT Inhibition

**Figure 7:**
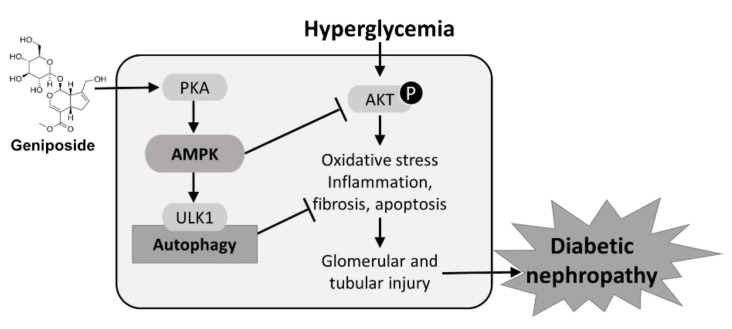
A schematic diagram illustrating protective mechanisms of geniposide against DN. Geniposide enhances autophagy and inhibits oxidative stress through AMPK activation and AKT inhibition. AMPK activity stimulated by geniposide increases ULK1-mediated autophagy and inhibits hyperglycemia-induced AKT signaling pathway, thus ameliorating glomerular and tubular injury and the progression of DN.

Hyperglycemia-induced AKT phosphorylation promotes autophagy dysfunction and accumulation of oxidative stress, which results in glomerular and tubular injury, renal inflammation, apoptosis and interstitial fibrosis that contribute to the development of DN. Geniposide treatment enhances ULK1-mediated autophagy response through the activation of PKA and AMPK pathways, and inhibition of AKT, thereby attenuating the pathogenesis of DN (Figure 7). Geniposide serves as a potential renoprotective agent by delaying the development and progression of DN.

## 3. Discussion

The present study investigated therapeutic effects of geniposide in DN mice, and the results demonstrated that geniposide significantly reduced kidney structural and functional abnormalities. First, geniposide reduced plasma creatinine, albuminuria, podocyte loss and glomerular and tubular injury. Second, geniposide attenuated interstitial fibrosis by decreasing collagen accumulation and fibrogenic gene expression in diabetic kidney. Third, geniposide suppressed renal apoptosis and oxidative stress, which are associated with induction of PGC-1α and antioxidant genes. Fourth, geniposide reduced the expression of pro-inflammatory cytokines and chemokines, glomerular macrophage infiltration in diabetic kidney. We propose that a renoprotective mechanism of geniposide is triggered by inducing autophagy response through activating AMPK pathway to reverse the progression of DN. Particularly, geniposide activates AMPK for induction of autophagy through ULK1 pathway, while inhibiting AKT activation, to suppress oxidative stress, thereby improving renal apoptosis, inflammation and interstitial fibrosis in DN.

Conventionally, glomerular hyperfiltration estimated by albuminuria or elevated plasma creatinine, is considered as the primary pathogenic driver of DN; however, proximal tubulopathy also contributes to the progression of DN due to tubular hypoxia caused by high energy demand, mitochondria dysfunction and peritubular capillary loss [47,48]. Empagliflozin, a recently published therapeutic drug targeting proximal tubules, is a selective inhibitor of sodium-glucose cotransporter 2 (SGLT2) and significantly reduced the progression of kidney disease in type 2 diabetic patients [49]. Proximal tubular injury progresses to interstitial fibrosis due to maladaptive repair, potentially inducing secondary glomerulosclerosis; this was supported by a study using multiple insults of tubular specific-toxin that cause interstitial fibrosis and glomerulosclerosis, aggravating the pathogenic transition from acute kidney injury to CKD [50,51,52]. Consistently, we found that geniposide attenuated the expression of KIM-1 and NGAL (specific tubular injury markers [26]) and reduced interstitial fibrosis which was confirmed by a decrease in collagen deposition and fibrogenic gene expression in diabetic kidney. Furthermore, geniposide decreased tubular apoptosis induced by DN, indicating that geniposide plays an active role in reducing tubular damage, substantially ameliorating tubular dysfunction and serves as a promising therapeutic agent to slow the progression of DN. 

Chronic hyperglycemia increases oxidative stress, modifying proteins and lipids that induce glucoxidation and peroxidation [53]. Reactive oxygen species also contribute to microvascular complications of DN by promoting immune cells infiltration and secretion of various cytokines and chemokines, associated with endothelial dysfunction and glomerular and tubular injury [54,55,56]. The expression of 4-HNE, a major product of lipid peroxidation process, is elevated in diabetic kidney and stimulates multiple pathological activities. 4-HNE can covalently react with nucleophilic residues (cysteine, histidine and lysine) to induce protein dysfunction [57] and activate apoptotic factors through the extrinsic and intrinsic pathways [58]. The elevated 4-HNE products alter extracellular matrix proteins, induce macrophage activation and promote the release of inflammatory and vasoactive mediators, leading to excessive kidney damage [59,60]. In this study, we measured 4-HNE as a marker of oxidative lipid, but other markers of oxidative DNA and protein may strongly support the protective effect of geniposide against oxidative damages induced in diabetic kidney [61]. The redox imbalance in diabetic kidney leads to the development of albuminuria, glomerular and tubular injury, renal hypertrophy, chronic inflammation and interstitial fibrosis [54,55,62]. Consistently, our results suggest that the antioxidant effect of geniposide are associated with a significant reduction in renal apoptosis, inflammation and interstitial fibrosis, ultimately improving renal pathology and function in DN. 

Recent research suggests that elevated uric acid levels are associated with the progression of chronic renal diseases, where uric acid promotes the activation of RAAS and increases the risk of reduced glomerular filtration rates [63,64]. Uric acid in tissues acts as a strong pro-oxidant, thus treatment with urate-lowering drugs (e.g., allopurinol) has shown to prevent glomerular endothelial dysfunction and the associated pathology of DN [65,66]. Dietary antioxidant supplements (e.g., vitamins C and E, polyphenols, flavonoids, etc.) in combination with other standard therapies can synergistically alleviate the progression of DN, by effectively reducing the uric acid-derived oxidative damage [67,68,69].

Autophagy is an essential adaptive cellular response to the metabolic stress in DN, and autophagy deficiency is strongly implicated in the progression of DN [6,70]. Pharmacological activation of autophagy restores autophagy activity and removes dysfunctional proteins and organelles, exerting an important renoprotective mechanism against DN [13,71]. In this study, we found that geniposide enhanced autophagy activity, which was suppressed in DN, by increasing autophagy flux (as assessed by LC3B-II and p62), upregulating autophagy-related proteins (ATG5, ATG12 and Beclin-1), and stimulating ULK1-mediated autophagy activity. Then, we investigated several signaling kinases including AMPK, PKA, AKT and GSK3β that are known to mediate autophagy response, hyperglycemia-induced oxidative stress and/or apoptosis in diabetic kidneys. 

AMPK is a nutrient-sensing kinase and critical regulator of autophagy induction. Under glucose starvation, AMPK promotes autophagy by directly activating ULK1 through the phosphorylation of Ser317 and Ser777. In contrast, mTOR or AKT activity at high glucose inhibits ULK1 activation by phosphorylating ULK1 at Ser757, thereby disrupting the interaction between ULK1 and AMPK and reducing the AMPK downstream signaling for autophagy induction [9,14,40,72]. Previous studies suggest that pharmacological activation of AMPK by AICAR or metformin significantly improves renal injury in diabetic mice by restoring autophagy activity [11,12]. Thus, AMPK-mediated autophagy induction is definitely involved in the renoprotection against DN. In this study, we demonstrated that geniposide enhanced autophagy by inducing AMPK phosphorylation and suppressing inhibitory phosphorylation of ULK1 (ser757). Geniposide also reduced the AKT activity, possibly enhancing autophagy response through ULK1 pathway. In addition, AMPK is activated by PKA through cAMP-mediated signaling through glucagon-like peptide-1 (GLP-1) receptor, where geniposide serves as an agonist of GLP-1R in the diabetic kidneys [41,73,74]. Consistently, we found that geniposide increased the activity of PKA, an upstream kinase of AMPK, possibly through GLP-1R activation, observed in the kidney of geniposide-treated DN mice (Figure 6B). 

GSK3β regulates various cellular processes including glycogen metabolism, cell cycle progression, apoptosis and cytoskeletal regulation [75]. Previously, high glucose and insulin were shown to induce GSK3β inactivation by phosphorylation at Ser9, which relies on activation of AKT to cause renal hypertrophy and extracellular matrix synthesis in proximal tubular cells and db/db mice [76]. Activation of GSK3β also ameliorates renal injury in STZ-induced type 1 diabetes [45]. Conversely, renal expression and activity of GSK3β are amplified in urinary exfoliated cells and diabetic patients, where the activated form of GSK3β at Tyr216 was elevated, predicting the progression of DN [44]. In this study, we demonstrated that GSK3β was significantly activated by geniposide in DN mice, as indicated by reduced levels of inactive p-GSK3β (Ser9), but not being altered in the active p-GSK3β (Tyr216) levels (Figure 6B). The result suggests that AKT-mediated GSK3β inactivation may play a prominent role in inducing renal hypertrophy and fibrosis observed in our DN mouse model. Interestingly, podocyte GSK3β is evolutionarily conserved to regulate the kidney function, and mice with podocyte-specific insulin resistance develop albuminuria and glomerular lesions [77,78], suggesting that GSK3β dysregulation plays a critical role in the pathogenesis of DN. Taken together, geniposide improves DN by enhancing ULK1-mediated autophagy and reducing oxidative stress, inflammation and fibrosis through AMPK activation. However, a couple of limitations occur in the current study to hold an immediate therapeutic application in DN patients. First, it is necessary to clarify the renal cell-type specific effects of geniposide to accurately treat different types of renal pathology. Second, the potential receptors for geniposide have not yet been determined other than confirming the expression of GLP-1R in the diabetic kidney. Third, the additional downstream effectors of p-AKT and p-GSK3β should be presented to validate the protective role of geniposide against DN. We propose to investigate the effect of geniposide on several kidney cell types—endothelial, glomerular, and tubular cells to determine the differential effects of geniposide. Cells treated with siRNA can be utilized to confirm the geniposide action through GLP-1R. There are also specific inhibitors available for signaling kinases that can be applied to demonstrate detailed pathways of geniposide.

In summary, we demonstrated that geniposide attenuates the pathological features of DN by improving albuminuria, podocyte loss, glomerular and tubular injury and fibrosis through the autophagy induction. The geniposide effects exerts through the activation of AMPK and suppression of AKT activity, suggesting geniposide as an effective therapeutic agent for DN.

## 4. Materials and Methods

### 4.1. Experimental Animals

Wild-type C57BL/6 male mice (7-week old) were purchased from Koatech Co. (Pyeongtaek, Korea) and maintained in the animal facility at Gyeongsang National University. All animal experiments were approved by the Institutional Board of Animal Research at Gyeongsang National University and performed according to the National Institutes of Health guidelines for laboratory animal care. Mice were housed with an alternating 12-h light/dark cycle and provided with water and standard chow ad libitum. 

### 4.2. DN Animal Model and Treatment 

We used a DN mouse model by combining unilateral nephrectomy (UNx), HFD and STZ treatment, as previously described [39]. Briefly, mice were habituated for 1 week and subjected to UNx. After 2 days, the mice were fed with a normal chow diet or a HFD (60 kcal% fat; Research Diets, Inc., New Brunswick, NJ, USA). After 3 weeks, a single dose of STZ (100 mg/kg) was intraperitoneally injected to HFD-fed mice. After STZ injection, the mice were treated with geniposide (50 mg/kg, daily) or vehicle (water) by oral gavage for 5 weeks. The control mice were fed with a normal chow diet (NCD) and received vehicle or geniposide. Geniposide was purchased from Sigma-Aldrich (St. Louis, MO, USA) and the dose of 50 mg/kg was selected based on the previous studies [79,80]. Hyperglycemia was assessed by measuring fasting blood glucose levels from the tail vein using an Accu-Check glucometer (Roche Diagnostics, Mannheim, Germany). After 5 weeks of geniposide treatment, all mice were sacrificed, and the right kidney was removed and weighted. Each half of kidney was snap-frozen in liquid nitrogen for storage at −80 °C or fixed in 10% buffered formalin for further analysis. Blood was collected from an inferior vena cava using a heparinized syringe and then centrifuged at 3000× *g* for 20 min, and the supernatants were stored at −80 °C for biochemical analysis. 

### 4.3. Biochemical Assays

Plasma creatinine was measured by a direct colorimetric Jaffe method and detected using a spectrophotometer (Shimadzu UV-1800 spectrophotometer, Tokyo, Japan), as previously described [39]. Urine samples were collected from each mouse housed in a metabolic cage (Jeungdo Bio & Plant Co., Seoul, Korea) for 15 h before sacrifice. Urine volume from each mouse was measured and centrifuged at 2000× *g* for 10 min to precipitate the sediments. Then the supernatant was transferred into a sterile tube for storage at −80 °C. Urine albumin and creatinine were determined by commercial assay kits from Abcam (Cambridge, MA, USA) according to the manufacturer’s instructions. Urine albumin excretion was presented by urine albumin-to-creatinine ratio (UACR), a ratio calculated by urine albumin being divided by absolute urine creatinine levels.

### 4.4. Periodic Acid–Schiff (PAS) and Picro-Sirius Red Staining

The 10% formalin-fixed kidney tissues were embedded in paraffin and sectioned at 5 μm of thickness. Kidney sections were stained with PAS staining (Abcam) for histological analysis and Picro-Sirius Red staining (Abcam) for visualization of collagen deposition. All stainings were performed by following the standard protocols. All images were captured using a CKX41 light microscopy (Olympus, Tokyo, Japan). 

### 4.5. Kidney Histological Examination

Renal histological abnormalities were assessed semi-quantitatively as previously described [28]. Briefly, after PAS staining, the severity of the glomerular injury was evaluated in randomly selected fields at 400× magnification and graded as follows: grade 0 = normal, grade 1 = <25%, grade 2 = 25–50%, grade 3 = 50–75% and grade 4 = 75–100% of segmental lesions. At least 30 glomeruli per group (*n* = 3–5) were analyzed. Tubular damage was scored by calculating the percentage of tubules that display tubular cell necrosis, sloughing of tubular epithelial cells or loss of brush borders, cast formation and tubular dilatation as follows: 0 = none, 1= ≤10%, 2 = 11–25%, 3 = 26–45%, 4 = 46–75% and 5 = ≥75%. At least 15 random fields (400× magnification) per group were evaluated (*n* = 3–5). For Picro-Sirius Red staining, ten kidney sections from each group were analyzed (200× magnification) to calculate the fibrotic area (*n* = 3–5), as previously described [81], using the ImageJ software (National Institutes of Health (NIH), Bethesda, MD, USA). 

### 4.6. Immunohistochemistry (IHC) Analysis

The 10% formalin-fixed and paraffin-embedded kidney tissue sections (5 μm-thick) were prepared. Briefly, the fixed kidney sections were deparaffinized, rehydrated and antigen-retrieved in sodium citrate buffer (10 mM, pH 6.0) for 20 min. The sections were blocked in 10% normal horse serum and incubated with a primary antibody against CD68 (Cluster of Differentiation 68) from (Abcam) or Wilms’ Tumor Protein 1 (WT1) from Boster Biological Technology (Pleasanton, CA, USA) overnight at 4 °C. The sections were incubated with a biotinylated secondary antibody (Vector Laboratories, Burlingame, CA, USA) for 1 h at room temperature. The sections were incubated in avidin-biotin-peroxidase complex solution (ABC solution, Vector Laboratories) for 30 min and developed using 3,3′-diaminobenzidine (DAB) Peroxidase Substrate Kit (Vector Laboratories). Then, the sections were counterstained with Mayer’s hematoxylin and analyzed using a CKX41 light microscope (Olympus). The number of CD68-stained cells or WT1-stained nuclei (equivalent to the number of podocytes) was counted from 10 images of 400× magnification per kidney section from each group, (*n* = 3), using ImageJ software (NIH). 

### 4.7. Immunofluorescence (IF) Staining

Mouse kidneys were fixed in 10% formalin and 5 μm-thick sections were deparaffinized, rehydrated and permeabilized in sodium citrate buffer (10 mM, pH 6.0) for 20 min. The sections were blocked in 10% normal goat serum and labelled with a mouse primary antibody against p62 (Abcam) overnight at 4 °C. After washing, the sections were incubated with a goat anti-mouse secondary antibody conjugated with Alexa Fluor^®^ 594 (red) for 1 h at room temperature and mounted with ProLong Gold antifade mounting solution containing 4′,6-diamino-2-phenylindole (DAPI, Thermo Fisher Scientific, Waltham, MA, USA) (*n* = 3). The images were captured using an Olympus Fluoview FV1000 confocal microscope and analyzed using Olympus Fluoview FV1000 software (Olympus). 

### 4.8. Terminal Deoxynucleotidyl Transferase dUTP Nick-End Labeling (TUNEL) Assay 

TUNEL staining was performed to evaluate the degree of apoptosis using an In Situ Cell Death Fluorescein Detection Kit (Roche Molecular Biochemicals, Mannheim, Germany) according to the manufacturer’s instructions. The images were captured using a Nikon Eclipse Ti-U microscope (Tokyo, Japan). The number of TUNEL-positive cells/high power field (HPF) was counted from 5 images of 200× magnification per section from each group (*n* = 3) using ImageJ software (NIH). 

### 4.9. Western Blot Analysis

Kidney tissues were homogenized in ice-cold radio-immunoprecipitation assay (RIPA) buffer with protease inhibitors (Thermo Fisher Scientific), sonicated and incubated for 20 min on ice. After centrifugation, the supernatant was transferred to a clean tube, and the protein concentration was determined using a PierceTM bicinchoninic acid (BCA) protein assay kit (Thermo Fisher Scientific). The protein lysates were separated using sodium dodecyl sulfate-polyacrylamide gel electrophoresis (SDS-PAGE), transferred to polyvinylidene difluoride (PVDF) membranes and blocked with 5% skim milk or 3% bovine serum albumin (BSA). The membranes were incubated with primary antibodies against AKT, p-AKT, AMPK, p-AMPK, autophagy-related gene 5 (ATG5), ATG12, Beclin-1, Light-chain 3B (LC3B), p62, uncleaved caspase-3, cleaved caspase-3, uncleaved poly (ADP-ribose) polymerase 1 (PARP-1), cleaved PARP-1, PKA Cα, and p-PKA Cα (from Cell Signaling Technology, Danvers, MA, USA); ULK1, p-ULK1, PGC-1α, and 4-HNE (Abcam); GSK3β (Santa Cruz Biotechnology, Dallas, TX, USA); p-GSK3β (Invitrogen, Carlsbad, CA, USA); GLP-1R (Proteintech group, Chicago, IL, USA); and β-actin (Sigma) in the blocking solution at 4°C overnight. Next, the membranes were incubated with the appropriated horseradish peroxidase (HRP)-conjugated secondary antibodies (Bio-Rad, Hercules, CA, USA) at room temperature for 1 h and then visualized with the ECL substrate (Bio-Rad). The ChemiDoc XRS + System (Bio-Rad) was used to evaluate the density of protein bands, and relative protein levels were quantified using Image Lab^TM^ software (Bio-Rad). 

### 4.10. Quantitative Real-Time Polymerase Chain Reaction (PCR) Analysis

The total RNA was extracted with Trizol (Invitrogen) and converted into cDNA using the RevertAid Reverser Transcription System (Thermo Fisher Scientific) according to the manufacturer’s protocol. Real-time PCR analysis was performed with a CFX Connect real-time PCR System using iQ SYBR Green Supermix (Bio-Rad). Real-time PCR analysis was performed with initial denaturation at 94 °C for 5 min, and the cycling conditions were 45 cycles of 10 s at 95 °C, 10 s at 60 °C and 30 s at 72 °C. Relative mRNA levels were normalized to those of glyceraldehyde 3-phosphate dehydrogenase (GAPDH). The primer sequences are listed in Table 1.

### 4.11. Statistical Analysis

Statistical significance was determined using one-way analysis of variance (ANOVA), followed by Bonferroni’s multiple comparisons test. All statistical analyses were performed with GraphPad Prism 7 Software v.7.00 (GraphPad Software Inc., La Jolla, CA, USA). Data were expressed as the mean ± SEM. * *p* < 0.05, ** *p* < 0.01, *** *p* < 0.001 vs. control mice; and ^#^ *p* < 0.05, ^##^ *p* < 0.01, ^###^ *p* < 0.001 vs. DN mice.

## Figures and Tables

**Table 1:** The primer sequences used for real-time PCR analysis in this study.

Gene	Forward Primers (5′–3′)	Reverse Primers (5′–3′)
KIM-1	GAGAGTGACAGTGGTCTGTATTG	CCTTGTAGTTGTGGGTCTTCTT
NGAL	CACCACGGACTACAACCAGTTCGC	TCAGTTGTCAATGCATTGGTCGGTG
CTGF	CAGCTGGGAGAACTGTGTACGG	CACACTCCGATCTTGCGGTTGG
TGFβ	CGAAGCGGACTACTATGCTAAA	TCCCGAATGTCTGACGTATTG
FN1	TACGGAGAGACAGGAGGAAATA	CATACAGGGTGATGGTGTAGTC
COL I	AGACCTGTGTGTTCCCTACT	GAATCCATCGGTCATGCTCTC
COL IV	CTGCTCTGCGTGGAGTATTT	AGGATGAAGGAGGCTAACAAAG
TIMP-1	CCAGTCATGGAAAGCCTCTGTGGA	CTTTGCTGAGCAGGGCTCAGAGTA
MCP-1	ACCTTTGAATGTGAAGTTGA	CTACAGAAGTGCTTGAGGTG
IL-6	CCAATTCATCTTGAAATCAC	GGAATGTCCACAAACTGATA
TNFα	CATATACCTGGGAGGAGTCT	GAGCAATGACTCCAAAGTAG
MIP2	AGAGGGTGAGTTGGGAACTA	GCCATCCGACTGCATCTATT
NOS2	GGAATCTTGGAGCGAGTTGT	CCTCTTGTCTTTGACCCAGTAG
IL-10	GGGAAGACAATAACTGCAC	TGAAAGAAAGTCTTCACCTG
NQO1	ATGACATCACAGGTGAGCTGAAGG	CTCAAACCAGCCTTTCAGAATGGC
MnSOD2	CCACCGAGGAGAAGTACCACGAG	CTCCTTATTGAAGCCAAGCCAGCC
GPX1	GAGAAGTGCGAAGTGAATGGTGAG	CACACCGGAGACCAAATGATGTAC
UCP2	GCG TTC TGG GTA CCA TCC TAA C	GCG ACC AGC CCA TTG TAG AG
GAPDH	GTGGCAAAGTGGAGATTGTTG	TTGACTGTGCCGTTGAATTTG

## Data Availability

Not applicable.
